# Environmental variables serve as predictors of the invasive Asian longhorned tick (*Haemaphysalis longicornis* Neumann): An approach for targeted tick surveillance

**DOI:** 10.1371/journal.pone.0292595

**Published:** 2023-11-02

**Authors:** R. T. Trout Fryxell, T. Chavez-Lindell, R. A. Butler, A. Odoi

**Affiliations:** 1 Department of Entomology and Plant Pathology, University of Tennessee, Knoxville, Tennessee, United States of America; 2 Department of Biomedical and Diagnostic Sciences, University of Tennessee, Knoxville, Tennessee, United States of America; University of Kentucky College of Medicine, UNITED STATES

## Abstract

Since the 2017 discovery of established populations of the Asian longhorned tick, (*Haemaphysalis longicornis* Neumann) in the United States, populations continue to be detected in new areas. For this exotic and invasive species, capable of transmitting a diverse repertoire of pathogens and blood feeding on a variety of host species, there remains a lack of targeted information on how to best prepare for this tick and understand when and where it occurs. To fill this gap, we conducted two years of weekly tick surveillance at four farms in Tennessee (three *H*. *longicornis*-infested and one without) to identify environmental factors associated with each questing life stage, to investigate predictors of abundance, and to determine the likelihood of not collecting ticks at different life stages. A total of 46,770 ticks were collected, of which 12,607 *H*. *longicornis* and five other tick species were identified. Overall, abundance of *H*. *longicornis* were associated with spring and summer seasons, forested environments, relative humidity and barometric pressure, sunny conditions, and in relation with other tick species. The likelihood of not collecting *H*. *longicornis* was associated with day length and barometric pressure. Additional associations for different life stages were also identified and included other tick species, climatic variables, and environmental conditions. Here, we demonstrated that environmental variables can be useful to predict the presence of questing *H*. *longicornis* and provide ideas on how to use this information to develop a surveillance plan for different southeastern areas with and without infestations.

## Introduction

The Asian longhorned tick, *Haemaphysalis longicornis* Neumann, is an exotic and invasive tick species capable of transmitting a number of bacterial, viral, and protozoan pathogens, as well as causing allergies related to the consumption of red meat [1–5]. In 2017, this tick was first discovered in the United States (US) feeding on a sheep in New Jersey [6]. At the time of submission of this manuscript, it has been found in 19 states where many cow-calf producers operate [7]. Shortly after its presence was confirmed, Virginia and USDA scientists reported *Theileria orientalis* Ikeda recovered from field-collected ticks and demonstrated this tick’s ability to transmit the deadly protozoan to cattle, causing many cases of Theileriosis [8–10]. Additionally, field-collected specimens in Virginia were confirmed with Bourbon virus [11]. While this tick is a 3-host tick, meaning it feeds on a different host for each life stage, it is also a generalist tick since life stages have been collected feeding from a variety of companion animals, livestock, and wildlife [7, 12]. The tick reproduces by parthenogenesis; a form of asexual reproduction similar to aphids which makes vector control extremely difficult and further emphasizes that prevention and rapid detection are necessary for management of this tick. A combination of public health professionals, state veterinary offices, and wildlife officials seek information on how to best prepare for this tick and understand when and where it occurs.

The confirmation of *H*. *longicornis* as a vector of *T*. *orientalis* Ikeda resulting in Bovine Theileriosis cases in the US represents a new and emerging pest and disease threat for the US cattle industry [8, 9]. In Australia, Theileriosis causes greater than $100 million in cattle damages annually and symptoms including lifelong infections and possible recrudescence [13]. Clinical infection of cattle with *T*. *orientalis* Ikeda is similar to infection with *Anaplasma marginale* with symptoms including lethargy, icterus, abortion, and death. Cattle living with this infection have decreases in milk production and growth rate [13]. Mortality rates range from 5–90% in susceptible herds, and even more alarming, there is no approved treatment [13, 14]. In addition, this tick can be hyperendemic, meaning thousands of ticks can be found parasitizing a single animal, as in the reported deaths of five calves in Surry County, NC [15]. The discovery of *H*. *longicornis* followed by *T*. *orientalis* Ikeda-infected animals caused by the bite of infected *H*. *longicornis* in Virginia [9] has created a food biosecurity threat with critical implications. This is because tick-borne diseases have the potential for widespread health, welfare, and economic impacts that challenge the sustainability of the entire US cattle industry.

In 2019, we reported *H*. *longicornis* in Tennessee and later confirmed its presence in 10 East Tennessee counties [16]. Reports of *H*. *longicornis* collected in Virginia, Delaware, New Jersey, and Pennsylvania, indicate larvae quest August through October, nymphs quest May through June, and adults quest July through August [17–21]. Virginia researchers also investigated several environmental variables associated with the presence of *H*. *longicornis* and found average daily temperature, habitat type, and precipitation metrics were associated with questing populations [21]. Knowing these variables and discovering additional variables associated with populations can allow stakeholders to better understand when and where these species occur. Importantly, we can use these predictor variables to develop monitoring and surveillance plans for *H*. *longicornis* driven by relevant field-collected data.

To improve the understanding of the distribution and dispersal of *H*. *longicornis* in Tennessee, we established a tick surveillance network with multiple state and federal agencies. A major goal of the network was to learn how to prevent and detect *H*. *longicornis* at infested sites [16]. We quickly learned that partnering with individuals who work closely with animals provided us with not only ticks, but also tick-infested properties. Thus, in agreement with private-property owners, we conducted two years of weekly tick surveillance at four Tennessee cow-calf farms (three infested with *H*. *longicornis* and one uninfested) to identify environmental predictors associated with each questing life stage. We then used those identified predictors to develop targeted surveillance programs. Here, we test the hypothesis that environmental variables can be identified to predict the presence and abundance of questing *H*. *longicornis* at different life stages and then use these results to help guide the development of detection methods and surveillance plans for *H*. *longicornis* in southeastern areas with and without infestations.

## Methods

### Design, setting, and tick collection

Using a prospective recruitment approach, this cross-sectional study evaluated the abundance of multiple tick species collected at four privately owned farms in three eastern Tennessee counties during 2019 and 2020. At each farm, 100 m transects were identified, representing three habitat types (forest, forest-field edge, and open pasture). Each farm chose their own management options after being provided guidance [22]. Farm 1 significantly reduced their *H*. *longicornis* populations after three years by keeping a closed herd, using an insecticidal spray, mowing pastures monthly to knee height, and allowing us to collect ticks regularly; while the other farms either kept an open herd, did not use a spray, or only mowed pastures yearly [23]. At farm 1, there were twelve transects in year one and then fifteen transects in year two to account for the reduced tick populations. The remaining three farms each had nine transects established (three transects for every habitat type). No surveillance was collected in areas with wet or damp vegetation (e.g., after rain, morning dew). We dragged for ticks along transects with a corduroy drag and checked the cloth every 10m along each transect for the presence of any tick species or life stage [24]. All ticks found on the drag cloth were then stored in a tube containing 80% ethanol or RNA later; one tube was used for each transect. Larvae were collected with labeled lint roller sheets and stored in plastic bags. Once a site was identified and confirmed as infested with *H*. *longicornis*, we began to drag weekly from epidemiological weeks 20–40 and then every other week for epidemiological weeks 41–19. Ticks were identified to species and life stage using a dissecting microscope and dichotomous keys [25–31].

### Variable selection and acquisition

Potential environmental predictors of *H*. *longicornis* abundance (larval stage, nymph stage, females, and total collected) were considered for investigation. At the beginning of each transect, we used a Kestrel 3000 pocket weather meter-heat stress monitor (Scientific Sales Inc, Lawrenceville NJ USA) to measure environmental conditions for each collection event (transect). These continuous variables included temperature, relative humidity, barometric pressure, and wind speed. We also identified categorical variables including farm, county, daylength (number of minutes between sunrise and sunset), season based on the equinox, habitat (identified at the site as edge, field, or forest), observed sun cover (cloudy, partly cloudy, full sun), and the number of habitat-sharing ticks of different life stages, sexes, and species obtained during collection. These included counts of larval stage, nymphal stage, males, females, and total collected for the species *Haemaphysalis leporispalustris* Packard, *Amblyomma americanum* L., *Dermacentor albipictus* Packard, *Dermacentor variabilis* Say, and *Ixodes scapularis* L.

### Descriptive analysis

All statistical analyses were performed using STATA Version 16.1 [32]. Relative activity, similar to seasonality, of each *H*. *longicornis* life stage was calculated by determining the mean percent of ticks collected during each epidemiological week of the study. Normality of the continuous variables was assessed using the Shapiro-Wilk test, implemented in the STATA *swilk* command. The continuous variables included daylength, temperature, relative humidity, barometric pressure, wind speed, and total number of ticks collected. All assessed variables were non-normally distributed; thus, median and interquartile ranges were used as the measures of central tendency and dispersion, respectively. Distribution of the categorical variables (collection year, county, farm site, season, habitat, and sun cover at collection start) and their 95% confidence intervals were computed in (SAS/R).

### Investigation of predictors of abundance

A series of negative binomial models, each built in two steps, was used to investigate the predictors of *H*. *longicornis* abundance. First, the STATA *glm* command was used to investigate the univariable associations between each potential predictor and the outcome under a Poisson distribution, applying a relaxed alpha level of 0.10. Since there was evidence of significant overdispersion of all Poisson models (Pearson dispersion parameters for life stages: 1.79–173.82; *P* < 0.05), as well as excess zero counts of the outcome variables, zero-inflated negative binomial (ZINB) models were fit to the data using the STATA *zinb* command. The Poisson and ZINB models were compared using their Akaike Information Criterion (AIC) values, with the model having the lowest value considered the best fitting model. Potential predictor variables with *P* < 0.10 were considered for inclusion in the multivariable models. Potential predictor variables identified in either the negative binomial or logit portions of the univariable zero-inflated models were used to develop multivariable models for each *H*. *longicornis* life stage.

The second step in model building involved development of a multivariable ZINB model for each age/sex category using a manual backwards elimination process using a critical alpha of 0.05. The coefficients of all variables were reviewed at each step for evidence of confounding. In situations where the removal of a variable resulted in a change of 20% or more in the coefficients of any of the variables in the model, the removed variable was considered a confounder and retained in the model, regardless of its statistical significance. No biologically plausible two-way interaction terms were identified for testing in the models. Incidence risk ratios (IR) and their 95% confidence intervals were computed for all variables retained in the final negative binomial part of the zero-inflated models. Odds ratios (OR) and their 95% confidence intervals were computed for all variables retained in the final logit part of the zero-inflated models.

## Results

### Tick collections

A total of 46,770 ticks were collected from four farms during the two years of study. These ticks consisted of 33,482 *A*. *americanum* (31,983 larvae, 1,329 nymphs, 106 males, and 64 females), 12,607 *H*. *longicornis* (7,833 larvae, 4,498 nymphs, 0 males, and 276 females), 461 *D*. *albipictus* (460 larvae and 1 nymph), 148 *D*. *variabilis* (42 larvae, 7 nymphs, 43 males, and 56 females), 67 *I*. *scapularis* (4 larvae, 24 nymphs, 25 males, and 14 females), and 5 *H*. *leporispalustris* (1 larva and 4 nymphs).

**Fig 1:**
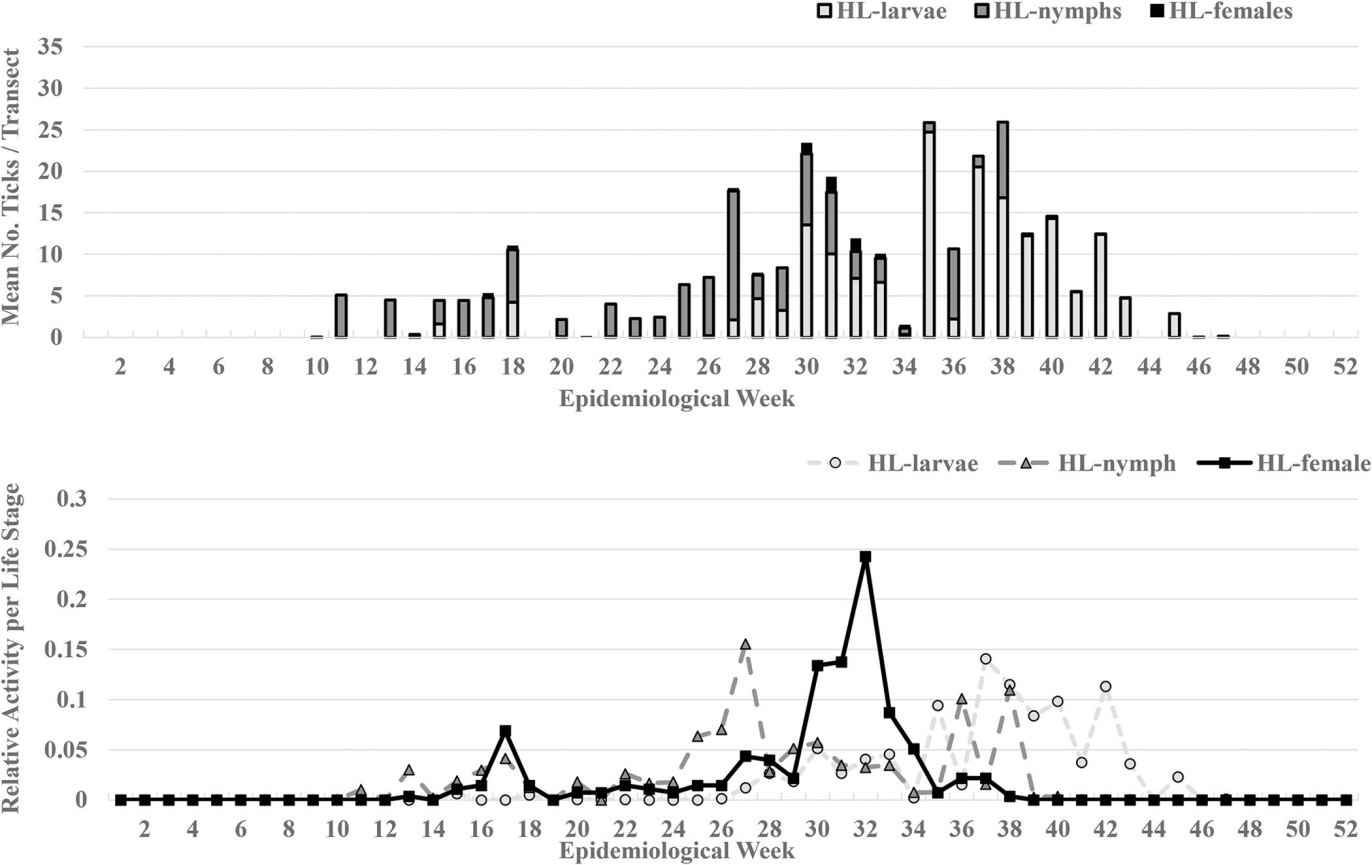
Collection data of *Haemaphysalis longicornis* (Hl) larvae, nymphs, and females presented as the mean number of each life stage collected (top) and relative activity by epidemiological week (bottom) on eastern Tennessee farms (2019–2020).

**Fig 2:**
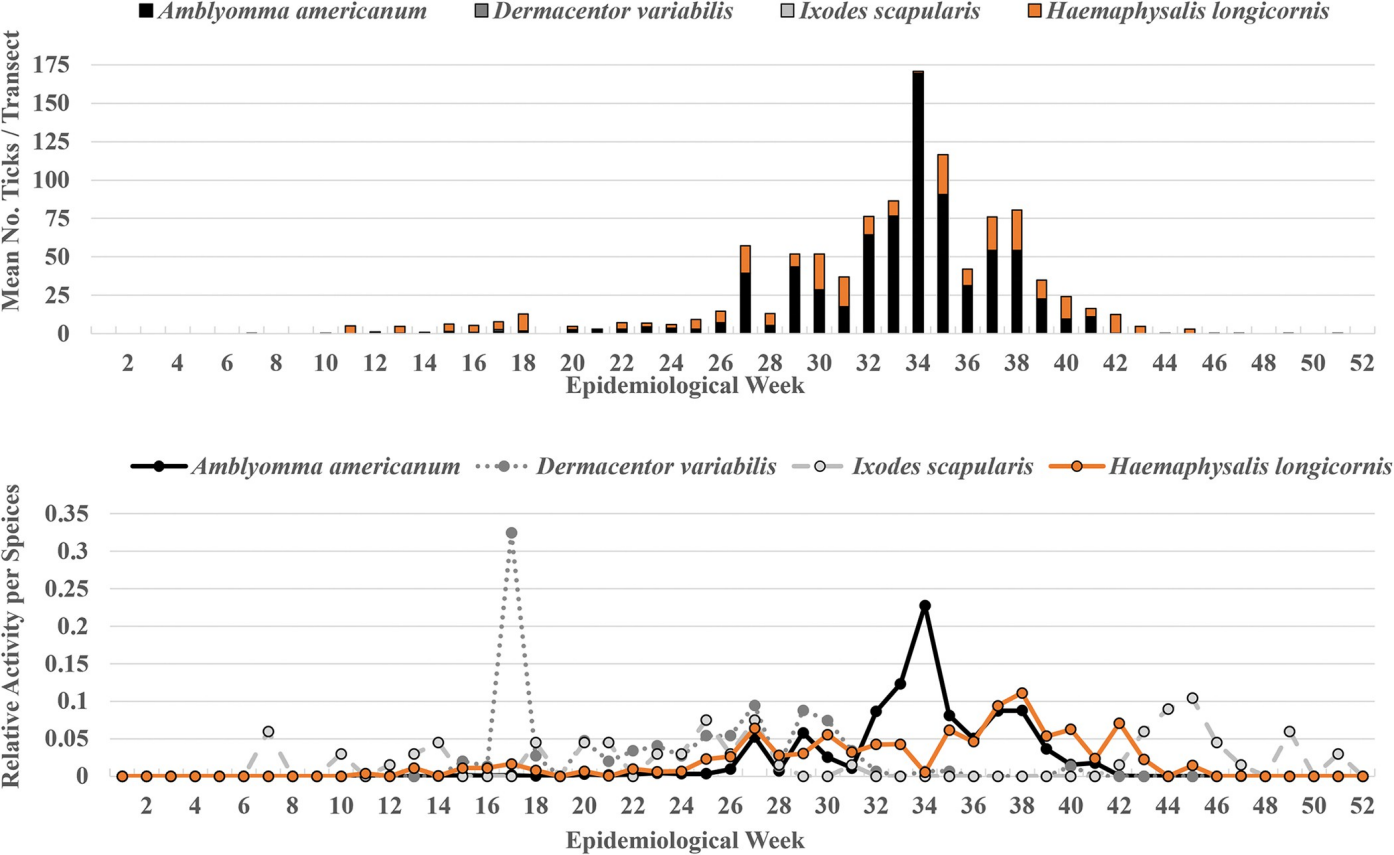
Collection data of *Amblyomma americanum*, *Dermacentor variabilis*, *Ixodes scapularis*, and *Haemaphysalis longicornis* as the mean number of each species collected and relative activity by epidemiological week on eastern Tennessee farms (2019–2020).

From the 1,497 100m-drags conducted during the study, a mean of 31.24 (median = 0) ticks were collected from each transect. A majority of the transects did not have any ticks (57.4%), but 637 collection events produced at least one tick; one transect produced 2,715 ticks. We collected *H*. *longicornis* at 341 transects with a mean of 8.42 per transect and a maximum of 602 *H*. *longicornis* ticks from one transect. Of the *H*. *longicornis* a mean of 5.23 larvae, 3.00 nymphs, and 0.184 females were collected per drag. The mean number of *H*. *longicornis* per epidemiological week and percent relative activity for each life stage indicated larvae were active from weeks 15 through 45 (peak 37–42), nymphs were active from weeks 11 through 38 (small peak 29–33 and larger peak 37–43), and females were active weeks 15 through 37 (peak 30–33) (**Fig 1**). For the remaining tick species, there were 362 instances of *A*. *americanum* (mean = 22.37; median = 0; max = 2715), 71 instances of *D*. *variabilis* (mean = 0.10; median = 0; max = 45), 55 instances of *I*. *scapularis* (mean = 0.04; median = 0; max = 4), 5 instances of *H*. *leporispalustris* (mean = 0; median = 0; max = 2), and 3 instances of *D*. *albipictus* (mean = 0.31; median = 0; max = 342) (**Fig 2**). Because collections of *H*. *leporispalustris* and *D*. *albipictus* were few and rare, we did not incorporate them as predictors or calculate relative activities for them.

Descriptive variables for tick counts (**Table 1**) and climatic data (**Table 2**) are presented. Six tick species were collected at Farm 1, but specimens were primarily *A*. *americanum*. Farm 2 was primarily infested with *H*. *longicornis* (99.62%), but three other species were also present in low numbers (*A*. *americanum*, *D*. *variabilis*, and *I*. *scapularis*). Farm 3 sampling began in 2020 and had five of the six tick species; most of these ticks were *H*. *longicornis*. Farm 4 did not have *H*. *longicornis* ticks but did have the remaining five species.

**Table 1:** Descriptive statistics (mean, standard deviation, range, and quartiles) for each tick species and life stage collected from four farms in eastern Tennessee (2019–2020).

Descriptive Variables		Ticks Collected per Drag			
		Mean ± Standard Deviation (Range)			
		Quartiles: 50%, 75%, 95%, 99%			
		Farm 1 (n = 723)	Farm 2 (n = 297)	Farm 3 (n = 81)	Farm 4 (n = 396)
*Haemaphysalis longicornis*	Larvae	6.18 **±** 32.706	6.34 **±** 23.765	18.31 **±** 59.522	0 **±** 0
		(0–383)	(0–174)	0–426	(0)
	Nymphs	2.96 **±** 26.844	7.39 **±** 26.682	1.98 **±** 5.24	0 **±** 0
		(0–602)	(0–346)	(0–36)	(0)
	Females	0.27 **±** 1.411	0.26 **±** 0.935	0.07 **±** 0.307	0 **±** 0
		(0–20)	(0–9)	(0–2)	(0)
	**Total**	**9.36 ± 43.230**	**13.95 ± 39.328**	**20.36 ± 60.893**	**0 ± 0**
		**(0–602)**	**(0–346)**	**(0–439)**	**(0)**
		50%: 0	50%: 0	50%: 0	50%: 0
		75%: 1	75%: 7	75%: 10	75%: 0
		95%: 45.6	95%: 83.20	95%: 124.2	95%: 0
		99%: 213.28	99%: 221.04	99%: 284.6	99%: 0
*Amblyomma americanum*	Larvae	10.28 **±** 75.683	0 **±** 0(0)	7.35 **±** 39.741	60.65 **±** 263.253
		0–1477		(0–280)	(0–2700)
	Nymphs	0.46 **±** 1.658	0.07 **±** 0.116	0.53 **±** 1.517	2.40 **±** 6.734
		(0–20)	(0–2)	(0–8)	(0–45)
	Males	0.05 **±** 0.276	0 **±** 0	0.12 **±** 0.620	0.15 **±** 0.617
		(0–4)	(0)	(0–5)	(0–8)
	Females	0.03 **±** 0.204	0 **±** 0	0.11 **±** 0.388	0.08 **±** 0.355
		(0–20)	(0)	(0–2)	(0–3)
	**Total**	**10.83 ± 75.841**	**0.07 ± 0.116**	**8.11 ± 39.652**	**58.30 ± 259.747**
		**(0–1482)**	**(0–2)**	**(0–280)**	**(0–2715)**
		50%: 0	50%: 0	50%: 0	50%: 0
		75%: 1	75%: 0	75%: 1	75%: 3
		95%: 20	95%: 0	95%: 12	95%: 370
		99%: 337	99%: 0	99%: 280	99%: 1451
*Dermacentor albipictus*	Larvae	0 **±** 0	0 **±** 0	0 **±** 0	1.16 ± 18.166
		(0)	(0)	(0)	(0–342)
	Nymphs	0 **±** 0	0 **±** 0	0 **±** 0	0 **±** 0
		(0)	(0)	(0)	(0)
	Males	0 **±** 0	0 **±** 0	0 **±** 0	0.003 ± 0.0503
		(0)	(0)	(0)	(0–1)
	Females	0 **±** 0	0 **±** 0	0 **±** 0	0 **±** 0
		(0)	(0)	(0)	(0)
	**Total**	**0.001± 0.0372**	**0 ± 0**	**0 ± 0**	**1.16 ± 18.166**
		**(0–1)**	**(0)**	**(0)**	**(0–342)**
		50%: 0	50%: 0	50%: 0	50%: 0
		75%: 0	75%: 0	75%: 0	75%: 0
		95%: 0	95%: 0	95%: 0	95%: 0
		99%: 0	99%: 0	99%: 0	99%: 0
*Dermacentor variabilis*	Larvae	0 **±** 0	0 **±** 0	0.49 ± 4.444	0.005 ± 0.1005
		(0)	(0)	(0–40)	(0–2)
	Nymphs	0.001± 0.0372	0 **±** 0	0.07 ± 0.468	0 **±** 0
		(0–1)	(0)	(0–4)	(0)
	Males	0.03 **±** 0.208	0.02 **±** 0.153	0.01 **±** 0.111	0.03 **±** 0.192
		(0–2)	(0–2)	(0–1)	(0–2)
	Females	0.04 **±** 0.249	0.02 **±** 0.163	0.10 **±** 0.436	0.04 **±** 0.222
		(0–3)	(0–2)	(0–3)	(0–2)
	**Total**	**0.07 ± 0.039**	**0.04 ± 0.251**	**0.68 ± 5.007**	**0.08 ± 0.317**
		**(0–4)**	**(0–3)**	**(0–45)**	**(0–2)**
		50%: 0	50%: 0	50%: 0	50%: 0
		75%: 0	75%: 0	75%: 0	75%: 0
		95%: 0	95%: 0	95%: 1	95%: 1
		99%: 2	99%: 1	99%: 45	99%: 2
*Ixodes scapularis*	Larvae	0 **±** 0	0 **±** 0	0 **±** 0	0.008 ± 0.1123
		(0)	(0)	(0)	(0–1)
	Nymphs	0.008 **±** 0.0908	0.003 ± 0.0580	0.04 ± 0.190	0.04 ± 0.264
		(0–1)	(0–1)	(0–1)	(0–4)
	Males	0.03 **±** 0.204	0 **±** 0	0 **±** 0	0 **±** 0
		(0–2)	(0–1)	(0)	(0)
	Females	0.02 **±** 0.133	0 **±** 0	0 **±** 0	0.005 **±** 0.0710
		(0–2)	(0–1)	(0)	(0)
	**Total**	**0.05 ± 0.247**	**0.01 ± 0.100**	**0.04 ± 0**	**0.05 ± 0.294**
		**(0–2)**	**(0–1)**	**(0–1)**	**(0–4)**
		50%: 0	50%: 0	50%: 0	50%: 0
		75%: 0	75%: 0	75%: 0	75%: 0
		95%: 0	95%: 0	95%: 0	95%: 0
		99%: 1	99%: 1	99%: 1	99%: 1
*Haemaphysalis leporispalustris*	Larvae	0 **±** 0	0 **±** 0	0 **±** 0	0.003 ± 0.0503
		0	(0)	(0)	(0–1)
	Nymphs	0.003 **±** 0.0744	0 **±** 0	0.01 **±** 0.111	0.003 ± 0.0503
		(0–2)	(0)	(0–1)	(0–1)
	Males	0 **±** 0	0 **±** 0	0 **±** 0	0 **±** 0
		(0)	(0)	(0)	(0)
	Females	0 **±** 0	0 **±** 0	0 **±** 0	0 **±** 0
		(0)	(0)	(0)	(0)
	**Total**	**0.003 ± 0.0744**	**0 ± 0**	**0.012 ± 0.111**	**0.005 ± 0.0710**
		**(0–2)**	**(0)**	**(0–1)**	**(0–1)**
		50%: 0	50%: 0	50%: 0	50%: 0
		75%: 0	75%: 0	75%: 0	75%: 0
		95%: 0	95%: 0	95%: 0	95%: 0
		99%: 0	99%: 0	99%:	99%: 62
**Total**		**20.39 ± 101.764**	**13.24 ± 39.177**	**29.20 ± 82.675**	**63.93 ± 264.642**
		**(0–2086)**	**(0–346)**	**(0–458)**	**(0–2715)**
		50%: 0	50%: 0	50%: 1	50%: 0
		75%: 3	75%: 3	75%: 18	75%: 4.5
		95%: 106	95%: 83	95%: 149	95%: 410
		99%: 388	99%: 220	99%: 458	99%: 1451
		**6 species**	**4 species**	**5 species**	**5 species**

**Table 2:** Descriptive statistics (mean, standard deviation, range, and quartiles) for climatic variables recorded at four farms in eastern Tennessee (2019–2020).

Potential Predictor Variables	Mean ± Standard Deviation (Range)			
	Quartiles: 25%, 50%, 75%			
	Farm 1 (n = 723)	Farm 2 (n = 297)	Farm 3 (n = 81)	Farm 4 (n = 396)
Day length (minutes)	764.01 **±** 89.499	747.30 **±** 106.275	772.33 **±** 66.564	758.45 **±** 78.378
	(583–875)	(583–876)	(656–855)	(583–875)
	25%: 695	25%: 633	25%: 724	25%: 696
	50%: 770.5	50%: 771	50%: 787	50%: 758.5
	75%: 854.5	75%: 853	75%: 827	75%:820.5
Temperature (°C)	24.49 **±** 7.881	23.32 **±** 9.985	26.87 **±** 6.165	22.70 **±** 6.627
	(1–39.5)	(1.33–43.5)	(14.5–41.6)	(1.5–36.2)
	25%: 21.70	25%: 15.60	25%: 21.50	25%: 18.44
	50%: 27.2	50%: 23.44	50%: 27.00	50%: 23.55
	75%: 29.90	75%: 31.823	75%: 31.55	75%: 27.5
Relative Humidity (%)	62.88 **±** 11.055	59.74 **±** 11.617	59.73 **±** 16.355	66.32 **±** 10.839
	(31.2–97.6)	(30.7–88.6)	(28.1–83.1)	(37.2–91.1)
	25%: 56.1	25%: 51.5	25%: 43.9	25%: 60.8
	50%: 63.05	50%: 60.9	50%: 62.1	50%: 67.7
	75%: 70.6	75%: 67.7	75%: 74.7	75%: 74.1
Barometric Pressure (mmHG)	29.15 **±** 0.103	28.77 **±** 0.133	28.81 **±** 0.101	29.10 **±** 0.119
	(29–29.4)	(28.4–29.1)	(28.2–28.9)	(28.9–29.4)
	25%: 29.10	25%: 28.70	25%: 28.8	25%: 29
	50%: 29.12	50%: 28.80	50%: 28.8	50%: 29.1
	75%: 29.20	75%: 28.84	75%: 28.9	75%: 29.2
Wind speed (m/s)	1.84 **±** 1.712	0.93 **±** 1.100	1.21 **±** 1.804	1.46 **±** 1.285
	(0–11.8)	(0–6.4)	(0–9.9)	(0–6.3)
	25%: 0	25%: 0.00	25%: 0	25%: 0
	50%: 1.6	50%: 0.80	50%: 0.8	50%: 1.3
	75%: 2.8	75%: 1.60	75%: 1.8	75%: 2.2

### Predictors of *Haemaphysalis longicornis* abundance

#### Univariable models

Potential predictors identified in univariable association with *H*. *longicornis* abundance (**Table 3**) included a range of climatic variables and habitat-sharing tick species, principally *A*. *americanum* and *I*. *scapularis*. The largest number of potential predictors was identified for nymphal stage *H*. *longicornis* abundance (13 variables) while just five potential predictors were identified for adult female *H*. *longicornis* abundance.

**Table 3:** Univariable associations of potential predictor variables for *Haemaphysalis longicornis* by life stage on four farms in eastern Tennessee (2019–2020). Significant values are bolded (*P* < 0.05).

Potential predictor variables		*Haemaphysalis longicornis*						
		Unadjusted Incidence Rate Ratio 95% Confidence Interval (p-value)						
		Larvae	Nymphs		Females		Total	
**Negative Binomial Part of the Zero-Inflated Model**								
Season (Overall)		*		**(<0.001)**		*		**(<0.001)**
Spring (vs. Fall)				**3.44, 36.35 (<0.001)**				**0.12, 0.46 (<0.001)**
Summer (vs. Fall)				**9.26, 91.59 (<0.001)**				0.58, 2.06 (0.774)
Winter (vs. Fall)				0.74, 28.73 (0.102)				**0.02, 0.44 (0.003)**
Daylength		1.00, 1.01 (0.089)		**1.00, 1.01 (0.04)**		**0.97, 1.00 (0.049)**		0.99, 1.00 (0.257)
Habitat (Overall)		**--**		**--**		**--**		**--**
		(0.849)		**(<0.001)**		**(0.042)**		(0.086)
Edge (vs. Field)		0.59, 2.53 (0.598)		**9.52, 66.48 (<0.001)**		**1.33, 23.92 (0.019)**		0.67, 2.71 (0.406)
Forest (vs. Field)		0.56, 2.74 (0.604)		**16.28, 134.82 (<0.001)**		0.69, 15.48 (0.138)		**1.01, 4.86 (0.046)**
Start Temperature		1.02, 1.13 (0.008)		0.95, 1.07 (0.806)		0.92, 1.06 (0.731)		0.99, 1.08 (0.147)
Relative Humidity		0.99, 1.05 (0.271)		**1.01, 1.05 (0.001)**		**1.01, 1.05 (0.008)**		**1.01, 1.05 (0.001)**
Barometric Pressure		0.10, 2.60 (0.426)		**0.00, 0.00 (<0.001)**		*		**0.03, 0.55 (0.005)**
Sun Cover (Overall)		**--**		**--**		**--**		**--**
		(0.958)		**(<0.001)**		(0.687)		**(0.005)**
Full Sun (vs. Cloudy)		0.49, 2.33 (0.874)		**2.30, 15.46 (<0.001)**		0.18, 3.04 (0.678)		0.62, 3.43 (0.387)
Partly Cloudy (vs. Cloudy)		0.18, 9.84 (0.776)		0.53, 4.95 (0.395)		0.11, 2.60 (0.433)		0.12, 1.04 (0.059)
Wind Speed		0.72, 1.18 (0.501)		**0.40, 0.69 (<0.001)**		0.53, 1.35 (0.487)		*
*Amblyomma americanum*	Larvae	0.099, 1.00 (0.196)		1.00, 1.01 (0.096)		*		1.00, 1.01 (0.128)
	Nymphs	0.61, 1.00 (0.051)		*		*		*
	Males	**0.00, 0.00 (<0.001)**		0.84, 7.11 (0.103)		0.41, 1.64 (0.568)		0.44, 1.92 (0.829)
	Females	0.18, 2.87 (0.643)		0.19, 2.25 (0.505)		0.14, 2.05 (0.369)		0.22, 2.14 (0.521)
*Dermacentor albipictus*	Larvae	Ø		Ø		*		Ø
	Nymphs					Ø		
	Males					*		
	Females					Ø		
*Dermacentor variabilis*	Larvae	Ø		Ø		*		Ø
	Nymphs	Ø		Ø		*		Ø
	Males	0.30, 148.29 (0.229)		**2.22, 47.88 (0.003)**		**1.11, 16.28 (0.034)**		**1.13, 14.11 (0.032)**
	Females	**0.06, 0.70 (0.011)**		**1.49, 14.04 (0.008)**		0.63, 9.77 (0.197)		0.79, 5.15 (0.143)
*Ixodes scapularis*	Larvae	Ø		0.00, 61.86 (0.686)		0.03, 402.58 (0.606)		0.00, 207.89 (0.780)
	Nymphs	**0.02, 0.51 (0.005)**		0.13, 9.76 (0.906)		0.18, 4.16 (0.845)		0.10, 3.43 (0.545)
	Males	Ø		**0.00, 0.38 (0.010)**		*		**0.00, 0.10 (0.000)**
	Females	**0.00, 0.31 (0.008)**		Ø		*		**0.00, 0.35 (0.007)**
*Haemaphysalis leporispalustris*	Larvae	Ø		Ø		*		Ø
	Nymphs			0.00, 124.09 (0.880)		*		0.00, 237.00 (0.813)
	Males			Ø		Ø		Ø
	Females			Ø		Ø		Ø
**Logit Part of the Zero-Inflated Model**								
Season (Overall)		*		(0.019)		*		(0.369)
Spring (vs. Fall)				0.00, .				0.00, .
				(1.000)				(0.978)
Summer (vs. Fall)				**0.02, 0.41 (0.002)**				0.00, 1.42 (0.082)
Winter (vs. Fall)				0.06, 2.63 (0.349)				0.28, 5.37 (0.781)
Daylength		**1.00, 1.01 (0.002)**		**0.88, 0.96 (<0.001)**		**0.91, 0.96 (<0.001)**		**0.96, 0.99 (<0.001)**
Habitat (Overall)		**(0.019)**		(0.781)		(0.896)		(1.000)
Edge (vs. Field)		0.44, 1.18 (0.187)		0.01, 8.90 (0.482)		0.01, 21.81 (0.640)		0.00, .
								(0.982)
Forest (vs. Field)		**0.27, 0.80 (0.006)**		0.00, .		0.00, .		0.00, .
				(0.982)		(0.982)		(1.000)
Start Temperature		0.97, 1.02 (0.928)		**0.65, 0.90 (0.001)**		**0.82, 0.94 (<0.001)**		0.07, 1.03 (0.055)
Relative Humidity		0.97, 1.00 (0.137)		0.02, 63.86 (0.995)		0.95, 1.03 (0.482)		0.00, .
								(1.000)
Barometric Pressure		**3.11, 36.85 (<0.001)**		**1.88, 183.80 (0.012)**		*		**21.48, 477.60 (<0.001)**
Sun Cover (Overall)		**(0.014)**		(0.851)		(1.000)		(0.647)
Full Sun (vs. Cloudy)		0.38, 1.27 (0.235)		0.19, 2.50 (0.570)		0.00, .		0.00, 8.79 (0.351)
						(0.997)		
Partly Cloudy (vs. Cloudy)		**1.18, 23.45 (0.029)**		0.00, .		0.00, .		0.00, .
				(0.998)		(0.986)		(0.979)
Wind Speed		0.85, 1.19 (0.941)		0.36, 1.54 (0.424)		0.69, 3.92 (0.263)		*
*Amblyomma americanum*	Larvae	1.00, 1.00 (0.676)		0.00, 0.00 (0.999)		*		0.00, .
								(0.998)
	Nymphs	0.93, 1.29 (0.281)		*		*		*
	Males	0.00, 0.00 (0.977)		0.00, .		0.01, 50.43 (0.819)		0.00, .
				(0.985)				(0.997)
	Females	0.48, 2.34 (0.886)		0.00, .		0.00, .		0.00, .
				(0.999)		(0.997)		(0.997)
*Dermacentor albipictus*	Larvae	Ø		Ø		*		Ø
	Nymphs					Ø		
	Males					*		
	Females					Ø		
*Dermacentor variabilis*	Larvae	Ø		Ø		*		Ø
	Nymphs	Ø		Ø		*		Ø
	Males	0.54, 24.47 (0.184)		0.00, .		0.00, 25.18 (0.529)		0.00, .
				(0.999)				(0.998)
	Females	0.34, 2.36 (0.824)		0.00, .		0.00, .		0.00, .
				(0.999)		(0.999)		(0.998)
*Ixodes scapularis*	Larvae	Ø		0.00, .		0.00, .		0.00, .
				(1.000)		(0.999)		(0.997)
	Nymphs	0.08, 1.64 (0.186)		0.00, .		0.00, .		0.00, .
				(1.000)		(0.996)		(1.00)
	Males	Ø		0.00, .		*		0.00, .
				(1.000)				(0.999)
	Females	0.01, 10.80 (0.516)		Ø		*		0.00, .
								(1.000)
*Haemaphysalis leporispalustris*	Larvae	Ø		Ø		*		Ø
	Nymphs	Ø		0.00, .		*		0.00, .
				(1.000)				(0.996)
	Males	Ø		Ø		Ø		Ø
	Females	Ø		Ø		Ø		Ø

* No estimate--model failed to converge;--No overall point estimate generated for categorical variables; Ø Did not estimate due to lack of variability in data or all observations = 0

#### Multivariable models

Each multivariable life stage model consisted of two parts, negative binomial and logit portions (**Table 4**).

**Table 4:** Multivariable model results for predictors of *Haemaphysalis longicornis* counts and presences on four farms in eastern Tennessee (2019–2020). Significant values are bolded (*P* < 0.05).

Potential predictor variables		*Haemaphysalis longicornis*							
		Larvae		Nymphs		Females		Total	
		Adjusted IR^+^ (95% CI^&^)	p-value	Adjusted IR (95% CI)	p-value	Adjusted IR (95% CI)	p-value	Adjusted IR (95% CI)	p-value
**Negative Binomial Model**									
Season (Overall)				--	**<0.001**			--	**<0.001**
Spring (vs. Fall)				0.81 (0.13–4.92)	0.814			0.07 (0.04–0.15)	**<0.001**
Summer (vs. Fall)				3.24 (0.61–17.12)	0.166			0.51 (0.26–1.01)	0.052
Winter (vs. Fall)				0.98 (0.09–11.24)	0.987			0.03 (0.01–0.10)	**<0.001**
Daylength						0.98 (0.97, 0.99)	0.003		
Habitat (Overall)				--	**<0.0001**	--	**0.0001**	--	**<0.001**
Edge (vs. Field)				14.86 (6.92–31.92)	**<0.001**	5.05 (2.42–10.55)	**<0.001**	1.14 (0.62–2.09)	0.685
Forest (vs. Field)				54.78 (21.76–137.92)	**<0.001**	4.98 (2.15–11.52)	**<0.001**	7.62 (3.75–15.49)	**<0.001**
Start Temperature		1.07 (1.00–1.14)	**0.042**						
Relative Humidity				0.97 (0.94–0.99)	**0.009**	1.03 (1.01–1.05)	**0.001**		
Barometric Pressure				0.02 (0.00–0.41)	**0.011**				
Sun Cover (Overall)				--	**0.047**				
Full Sun (vs. Cloudy)				2.87 (1.21–6.84)	**0.017**				
Partly Cloudy (vs. Cloudy)				2.05 (0.76–5.56)	0.159				
*Amblyomma americanum*	Larvae			0.99 (0.98–1.00)	**0.013**				
	Nymphs	0.63 (0.34–1.16)	0.140*						
	Males	0.01 (0.00–0.26)	**0.004**						
*Dermacentor variabilis*	Males					2.58 (1.16–5.71)	**0.020**		
	Females			0.29 (0.13–0.67)	**0.004**				
**Logit Model**									
Season (Overall)				--	**0.002**				
Spring (vs. Fall)				0.04 (0.00–0.33)	**0.003**				
Summer (vs. Fall)				0.34 (0.11–1.05)	0.060				
Winter (vs. Fall)				0.25 (0.01–7.86)	0.429				
Daylength		1.01 (1.00–1.01)	**0.001**			0.94 (0.91–0.96)	**<0.001**		
Barometric Pressure				142.20 (1.75–11,561.70)	**0.027**			56.03 (18.50–169.60)	**<0.001**
Sun Cover (Overall)		--	**0.019**						
Full Sun (vs. Cloudy)		0.56 (0.29–1.07)	0.080						
Partly Cloudy (vs. Cloudy)		3.04 (0.65–14.27)	0.158						
*Ixodes scapularis* nymphs		0.13 (0.02–0.79)	0.026						

+ IR = Incidence Rate Ratio; & CI = Confidence interval;--No overall point estimate generated for categorical variables

* Retained in model due to confounding

The observed density of larval *H*. *longicornis* populations increased as collection start temperatures increased (IR = 1.07, *P* = 0.042), but decreased as *A*. *americanum* nymphs and adult males increased (IR = 0.63, *P* = 0.140; IR = 0.01, *P* = 0.004, respectively). The observed density of nymphal *H*. *longicornis* populations were predicted by habitat, with forest-field edge and forest habitats demonstrating particularly large increases (IR = 14.86, *P* < 0.001; 54.78, *P* < 0.001, respectively) in comparison to field habitats, and by both full sun (IR = 2.87, *P* = 0.017) and partly cloudy (IR = 2.05, *P* = 0.159) relative to cloudy sun cover. In contrast, the observed density of nymph populations decreased with increases in relative humidity (IR = 0.97, *P* = 0.009), barometric pressure (IR = 0.02, *P* = 0.011), and populations of larval *A*. *americanum* (IR = 0.99, *P* = 0.013) and female *D*. *variabilis* (IR = 0.29, *P* = 0.004). The observed density of nymph populations were additionally predicted by season (overall *P* < 0.001), with large population increases in the summer (IR = 3.24, *P* = 0.166) compared to fall.

The logit portion of the model is used to predict the absence of the ticks and to identify factors associated with no tick collection. Here the logit model was used to predict the odds of collecting no *H*. *longicornis* in a field drag, suggesting the absence of questing ticks at a given timepoint at the collection site. Variables that were significantly associated with absence of questing larvae were increasing day length (OR = 1.01, *P* = 0.001) and decreasing population of *I*. *scapularis* nymphs (OR = 0.13, *P* = 0.026). Additionally, sun cover (overall *P* = 0.019) was a significant predictor of larval absence, with collections occurring under full sun conditions having only half the odds (OR = 0.56, *P* = 0.080) of finding no questing *H*. *longicornis* larvae as those taking place under cloudy conditions. The absence of questing nymphal *H*. *longicornis* were associated with season (overall *P* = 0.002), with spring, summer, and winter seasons having lower odds of finding zero counts compared to fall (OR = 0.04, *P* = 0.003; OR = 0.34, *P* = 0.060; OR = 0.25, *P* = 0.429, respectively), as well as increasing barometric pressure (OR = 142.20, *P* = 0.027). The absence of questing adult female *H*. *longicornis* specimens was associated with only daylength (OR = 0.94, *P* < 0.001), while the odds of a zero count of any questing *H*. *longicornis* stage on a drag was positively associated with changes in barometric pressure (OR = 56.03, *P* < 0.001).

## Discussion

As *H*. *longicornis* populations continue to disperse west across the US, our results are useful first steps in developing a targeted monitoring and surveillance plan. Most three-host ticks, like *H*.*longicornis* are r-reproductive strategists spending a majority of their life off of the host in the environment either molting to the next life stage or questing for a host. Thus, these behaviors make them reliant on the environment to provide a safe place to molt and avoid desiccation or predation and to also attract potential hosts to that habitat [33–35]. While we know from surveillance data that most first of the year collections of *H*. *longicornis* occurred in March [7], we can now include more detailed environmental variables for targeted tick surveillance. Knowing when and where those other species are collected can help identify collection sites for *H*. *longicornis*.

Developing surveillance and monitoring plans for exotic and invasive species is challenging for a variety of reasons, but primary reasons we experienced included resource availability (time and people) and knowledge or training. This frustration is not new and was reported by others after the invasion and spread of West Nile virus, Zika virus, and tick-borne pathogens [36]. Here, our data provide useful details for creating such a plan and tailoring it to a variety of geographic settings. Specifically, locations with forest-field edge habitats were predicted to be best for collecting all three life stages. Surveillance plans should be implemented when daylength begins to increase and conclude when daylength decreases. Monitoring climatic variables such as temperature, barometric pressure, and relative humidity will help programs know when to attempt to collect ticks given the highest likelihood of finding them. Specifically, vector ecologists can expect to collect larvae in warming temperatures, nymphs when relative humidity and barometric pressures decrease, and females when relative humidity increases. If a tick surveillance plan is already established in an area, using entomological data regarding other ticks collected can also help to plan collections across space and time. This could be different in collection sites with varying climatic variables (e.g., northeast US) which likely explains seasonality reports in those locations. In Tennessee, increasing collections of *A*. *americanum* and *D*. *variabilis* populations were both associated with decreased number of *H*. *longicornis* collections. Specifically, as *A*. *americanum* nymphs and adult males increased, *H*. *longicornis* larvae decreased. Increases in populations of larval *A*. *americanum* and female *D*. *variabilis* was associated with fewer *H*. *longicornis* nymphs. Female *H*. *longicornis* populations decreased as *D*. *variabilis* populations increased. Thus, monitoring populations of one species may help programs plan to monitor other species as their range increases.

In areas where *H*. *longicornis* specimens have not yet been confirmed or where it is suspected to occur in the future, community engagement events such as tick blitzes, might help with monitoring and surveillance for this species [37–39]. In such activities, community members and stakeholders are asked to collect any and all ticks they encounter or collect from an accessible and available location and provide those collections to a researcher to better understand tick presence. The goal is to reduce the numbers of zero counts and maximize collection; therefore, our logit model results highlight optimal conditions for conducting a data-driven tick blitz. For instance, if we were to organize a tick blitz for similar farm areas east of the Mississippi River, we would begin to advertise it in the spring to reduce chances of not finding ticks. Knowing when and where life stages are likely not questing will help identify when and where they could be questing, hence, minimizing efforts in areas or times of day when ticks are not likely to be questing. Larvae and females were more likely to be questing on sunny days and not questing as day length increased (early summer). Nymphs were not likely to be collected in the fall when barometric pressure increased (spring), which could suggest overwintering in immature stages (e.g., as an engorged larvae or molted nymph). Using these data, we would recommend scheduling tick blitzes on a sunny day in a forest or forest-field edge habitat just after the summer solstice when day length begins to decrease (~epidemiological weeks 25–35 in Tennessee). This period would also be similar to the northern reports of *H*. *longicornis* in Virginia, Delaware, New Jersey, and Pennsylvania [17–21]. By providing clear instructions such as these, community scientists can begin to scout and find new and unknown populations of exotic and invasive species most effectively. These results can also be used by field veterinarians to help find *H*. *longicornis* on a farm.

Additionally, data presented here can be used to help plan monitoring strategies in different areas. Importantly, *I*. *scapularis* populations were also on these farms, but their populations did not predict *H*. *longicornis* populations. This may be due to the fewer numbers found on these four farms or potential interactions on hosts. Repeating this study in an area with more *I*. *scapularis* will help explain their relationship with *H*. *longicornis*. Notably, *I*. *scapularis* populations are expanding southward into our study sites, so continued surveillance may enhance our understanding of the two species’ interaction. Previous research at sites with larger *I*. *scapularis* populations and reduced *A*. *americanum* populations has also confirmed *H*. *longicornis* populations. With more data, sites infested with *I*. *scapularis* may also be sites at greater risk for *H*. *longicornis* populations. For instance, in the northeastern US, many health departments conduct surveillance for *I*. *scapularis* and may use this opportunity to also collect *H*. *longicornis* when they are active. This idea is supported by previous research efforts conducting surveillance for *I*. *scapularis* which also collects questing *H*. *longicornis* concurrently [12, 17]. Results from our logit model also demonstrated an increase in *I*. *scapularis* nymphs decreases the likelihood of a zero count and that a decrease in population would increase the likelihood of a zero count. Finally knowing that one or more tick species are present at the same site and together can also lead to interesting questions concerning competition, cooccurrence, host use allocation, and pathogen transmission.

Collections at four cow-calf farms led to useful information about an exotic and invasive tick species, but also has the ability to increase general awareness about all tick species in the area. Specifically, we can use these data to remind people that *H*. *longicornis* populations appear to have two seasons, an early summer season and an early fall season. Data presented here are also useful when attempting to identify ticks. For instance, these ticks were not associated with *D*. *variabilis* populations, but were associated with both *A*. *americanum* and *I*. *scapularis*. We can use the tick association data to tell people that *H*. *longicornis* populations may increase again after reports of *A*. *americanum* larvae (or seed ticks) tend to wane. Likewise, many veterinarians may notice an increase in *D*. *variabilis* populations on animals presenting to their clinics and we recommend that as they note this observation, they should remind their clients that *H*. *longicornis* might also be present, particularly at the edge of their yards. Providing reminders to owners to be diligent with use of animal tick control products and checking their animals for ticks is especially important because many people in Tennessee believe there is a single tick season (summer). Furthermore, many pet owners do not recognize that throughout the year and in each season, there are several different tick species more likely to be abundant, and that those species may be associated with a specific set of tick-borne diseases.

## Conclusions

Undoubtedly, the presence of *H*. *longicornis* as well as the confirmation of *T*. *orientalis* Ikeda genotype in multiple US states presents a new and emerging pest and pathogen threat for the North American cattle and associated-livestock industries. *H*. *longicornis* confirmation also warrants continued development of integrated pest management plans for livestock producers and other stakeholders. Historically, tick management has been difficult due to their complex life histories which includes a three-year life span with a majority of time spent off host in the environment [40–46]. Herein, we identify factors associated with finding ticks in the environment and provide a list of accessible and measurable variables that can be used to plan and monitor for exotic and invasive *H*. *longicornis* populations. Together, these variables can be used by health departments and vector biologists to develop and create surveillance campaigns that best fit their jurisdiction, as well as the development of targeted tick management programs. Likewise, community members and stakeholders can become aware of the different species and use experience with one species to prepare and/or respond to another. Additionally, results could be used to assess management plans before trying them in the field. This might include using these data as optimized control models to identify the best life stages to manage or identifying different plans for monitoring under different conditions. We demonstrated that environmental variables are useful for predicting the absence/presence and abundance of an exotic and invasive tick population and provide ideas on how to use this information to develop a surveillance plan for different areas with and without infestations. Considering that *H*. *longicornis* is one of many potential invasive tick species poised to enter and spread across the US, it is imperative to continue to establish routine surveillance programs to not only monitor for invasive species, but to use those results to rapidly understand their behavior, seasonality, and distributions, for when (not if) they arrive. This is an essential biosecurity need.
